# Comprehensive immune profiling and predictive modelling of paediatric acute hepatitis of unknown aetiology from a Spanish cohort

**DOI:** 10.3389/fimmu.2025.1599982

**Published:** 2025-06-24

**Authors:** Roberto Lozano-Rodríguez, Loreto Hierro, María José Quiles, Alejandro Pascual-Iglesias, Verónica Terrón-Arcos, Gema Muñoz-Bartolo, Esteban Frauca, Francisco J. Cueto, Cristina Calvo, Laura Córdoba-García, Jesús Fernández-Felipe, Laura Hurtado-Navarro, Julia del Prado-Montero, Gonzalo Sáenz de Santa María-Diez, Daniel Arvelo-Rosario, Paloma Jara, Carlos del Fresno, Eduardo López-Collazo

**Affiliations:** ^1^ The Innate Immune Response Group, Instituto de Investigación Hospital Universitario La Paz (IdiPAZ), La Paz University Hospital, Madrid, Spain; ^2^ Tumour Immunology Laboratory, Instituto de Investigación Hospital Universitario La Paz (IdiPAZ), La Paz University Hospital, Madrid, Spain; ^3^ Department of Paediatric Hepatology, Instituto de Investigación Hospital Universitario La Paz (IdiPAZ), La Paz Children’s University Hospital, Madrid, Spain; ^4^ Department of Paediatrics, Instituto de Investigación Hospital Universitario La Paz (IdiPAZ), La Paz Children’s University Hospital, Madrid, Spain; ^5^ Centre for Biomedical Research Network, Centro de Investigación Biomédica en Red (CIBER) of Infectious Diseases (CIBERINFEC), Madrid, Spain; ^6^ Biobank Platform, Instituto de Investigación Hospital Universitario La Paz (IdiPAZ), La Paz University Hospital, Madrid, Spain; ^7^ Centre for Biomedical Research Network, Centro de Investigación Biomédica en Red (CIBER) of Respiratory Diseases (CIBERES), Madrid, Spain; ^8^ Biomedical Department, UNIE Universidad, Madrid, Spain

**Keywords:** hepatitis, unknown aetiology, biomarker, Gal-9, sTim-3

## Abstract

**Introduction:**

Paediatric acute hepatitis of unknown aetiology (PAHUA) has emerged as a global health concern, yet its cause remains unidentified. This study characterises the clinical and immunological profiles of PAHUA to identify reliable immune biomarkers for accurate diagnosis.

**Methods:**

Samples from 24 PAHUA patients, 6 children with autoimmune hepatitis (AIH), and 13 healthy paediatric volunteers (HVs) were analysed. Immunophenotyping, soluble immune checkpoints (ICs) and cytokine profiling, and *ex vivo* immune responses were assessed using spectral flow cytometry. Clustering and logistic regression modelling were used to identify predictive biomarkers.

**Results:**

PAHUA cases frequently presented with gastrointestinal symptoms and liver damage preceding jaundice, with 59% progressing to paediatric acute liver failure (pALF). Adenovirus was detected in only 17.6% of PAHUA patients, suggesting it is unlikely to be the primary causative agent. Antibodies against the SARS-CoV-2 Spike protein were identified in 88.2% of PAHUA patients, as well as in AIH and HV groups, indicating prior exposure. Immunophenotyping, ICs and cytokine profiling, and *ex vivo* immune revealed distinct immune profiles between PAHUA and non-PAHUA individuals. Furthermore, clustering and logistic regression modelling identified potential predictive biomarkers, including the plasmatic ICs Gal-9 and sTim-3, alongside specific immune cell populations. Notably, a combined Gal-9 and sTim-3 model achieved an AUC of 1.000 in differentiating PAHUA patients from non-PAHUA individuals.

**Conclusions:**

Despite the limited cohort analysed, owing to the rarity of cases worldwide, our data provide valuable insights for an accurate, early, and minimally invasive diagnosis of PAHUA. These patients exhibit a distinct immunological profile, with ICs, particularly Gal-9 and sTim-3, showing strong potential as reliable biomarkers.

## 1 Introduction

Severe acute hepatitis of unknown origin has emerged as a critical global health concern, particularly in children, with cases reported worldwide. Unlike viral hepatitis caused by known agents (hepatitis A–E), the aetiology of paediatric acute hepatitis of unknown aetiology (PAHUA) remains unresolved. Initial cases were reported between October 2021 and February 2022 at the Children’s Hospital of Alabama, where affected children tested negative for known causes of hepatitis (1, 2). By April 2022, an outbreak in the United Kingdom led to a surge in cases globally, prompting a World Health Organization (WHO) alert (3, 4). As of July 2022, over 1010 probable cases, including 22 fatalities, had been reported worldwide, with the majority concentrated in Europe and the Americas (5, 6).

The WHO and European Centre for Disease Prevention and Control (ECDC) define probable PAHUA cases as acute hepatitis (non-A–E) with serum transaminase levels exceeding 500 IU/L in individuals aged ≤16 years since October 2021 (7, 8). Symptoms include gastrointestinal disturbances (vomiting and diarrhoea) followed by jaundice, without fever, and laboratory findings reveal elevated ALT and AST, hyperbilirubinemia, and coagulation abnormalities consistent with paediatric acute liver failure (pALF). While environmental toxins and known viruses have been ruled out, potential associations with adenovirus and SARS-CoV-2 remain speculative.

Despite considerable efforts to determine an unifying cause, the aetiology of PAHUA remains unresolved (5, 9). Proposed mechanisms include viral, toxic, and immune-mediated injury, though evidence is inconclusive (10–12). Given the rapid clinical deterioration in some patients, including progression to pALF and the requirement for transplantation, early diagnosis is critical.

This study aims to characterise the clinical and immunological profiles of PAHUA and to identify diagnostic and prognostic biomarkers. Acknowledging the limited cohort analysed and the rarity of cases worldwide, our findings provide valuable insights that may facilitate the classification of such patients in future occurrences. Utilising full-spectrum flow cytometry, we examined immune cell populations, cytokine profiles, and soluble immune checkpoints, identifying biomarkers with the potential to enhance the diagnosis and management of this paediatric liver disease.

## 2 Materials and methods

### 2.1 Patients and participants

**Figure 1:**
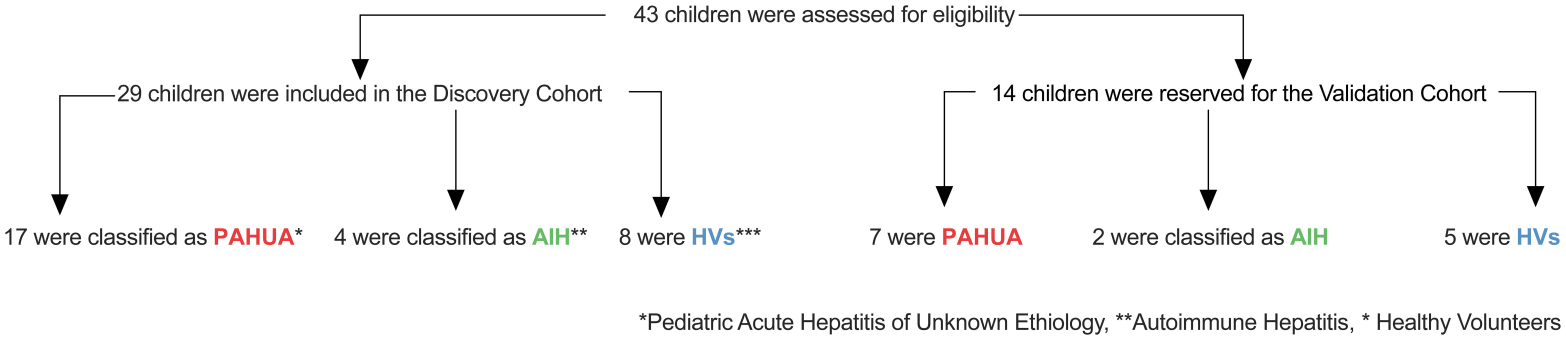
Enrolment of confirmed cases of PAHUE, AIH and HV cases in both the discovery and validation cohorts. PAHUA denotes Paediatric Acute Hepatitis of Unknown Aetiology, HV represents healthy paediatric volunteers, and AIH stands for autoimmune hepatitis.

Between May 2022 and January 2023, 43 participants (30 paediatric patients and 13 healthy volunteers) were recruited from the Paediatric University Hospital La Paz (Madrid, Spain). A discovery cohort of 29 participants included eight healthy paediatric volunteers (HVs) and 21 children with acute hepatitis. Of these, 17 patients met the ECDC/WHO criteria for PAHUA, which considers hepatitis virus serology, transaminase levels, and age (**Figure 1**). Four children were excluded due to confirmed alternative diagnoses of autoimmune hepatitis (AIH). The AIH group served as an internal control for conditions with similar clinical presentations. A validation cohort was established with 7 PAHUA patients, 2 AIH patients, and 5 HVs to confirm the predictive model. All blood samples were collected from the participants prior to treatment.

### 2.2 Antibodies and immunophenotyping by flow cytometry

Fresh blood from all participants was collected in EDTA-anticoagulated tubes (Fisher Scientific, Massachusetts, United States, #Cat: 10081844). Whole white blood cells were enriched following red blood cell lysis. Briefly, 2 mL of EDTA-anticoagulated venous blood were treated with 20 mL of 1X Pharm Lyse Buffer (BD Biosciences, New Jersey, United States, #Cat: 555899) for 15 minutes at room temperature (RT) on a rocker. After two PBS (Phosphate Buffered Saline, Sigma, Massachusetts, United States, #Cat: P4417-100TAB) washes, cells were stained with LIVE/DEAD Fixable Blue Dead Cell Stain Kit (Invitrogen, Massachusetts, United States, #Cat: L23105) to exclude dead cells. Prior to labelling, True-Stain Monocyte Blocker (BioLegend, California, United States, #Cat:426103) was added to prevent nonspecific binding of some fluorochromes to monocytes. Cells were then labelled with fluorochrome-conjugated monoclonal antibodies for 25 minutes at RT in the dark. Two separate cytometry panels (**Supplementary Tables S1**, **S2**) were used to analyse immune cell populations and immune checkpoint expression, respectively. Labelled cells were acquired using a Cytek Aurora Spectral Cytometer (Cytek Biosciences, California, United States). Two additional cytometry panels (**Supplementary Tables S3**, **S4**) were used to assess CD4/CD8 proliferation and memory profiles and monocyte phenotyping. Data were analysed using FlowJo software (v10.6.2, TreeStar).

### 2.3 PBMC isolation and culture conditions

Peripheral blood mononuclear cells (PBMCs) were extracted from the same EDTA-anticoagulated venous blood samples used for immunophenotyping in both paediatric patients and healthy paediatric volunteers. The isolation was performed using Ficoll-Plus (Cytiva, Massachusetts, United States, #Cat: 17144003) in accordance with the manufacturer’s instructions. The PBMCs were washed twice with phosphate-buffered saline (PBS), stained with Trypan blue, and counted. Freshly isolated PBMCs were cultured in RPMI 1640 medium (ThermoFisher Scientific, Massachusetts, United States, #Cat: 72400112) supplemented with 10% foetal bovine serum (FBS, ThermoFisher Scientific, Massachusetts, United States, #Cat: A5669701), 25 mM HEPES, 2 mM L-glutamine, and 1% Penicillin-Streptomycin (ThermoFisher Scientific, Massachusetts, United States, #Cat: 15140122) prior to stimulation to induce cellular activation or proliferation. The cultures were maintained at 37°C in a humidified incubator with 5% CO_2_.

### 2.4 Plasma collection

Plasma samples were collected from EDTA-anticoagulated venous blood using a Ficoll-Plus (Cytiva, Massachusetts, United States, #Cat: 17144003) solution, following standard density gradient centrifugation. The plasma samples were aliquoted and stored at -80°C until further use.

### 2.5 Quantification of anti-Spike IgA and IgG SARS-CoV-2 antibodies

Plasma samples were thawed, and centrifuged at 1000 RCF for 30 minutes to remove particulates before use. The titres of SARS-CoV-2-specific IgG antibodies against the Spike (S1 and receptor binding domain [RBD]) and Nucleocapsid (N) proteins were measured using the LEGENDplex SARS-CoV-2 Serological IgG Panel (Biolegend, California, United States, #Cat: 741132), following the manufacturer’s instructions. The samples were analysed using a FACSCalibur flow cytometer (BD Biosciences, New Jersey, United States), and data were processed using LEGENDplex software (v.8, Biolegend).

### 2.6 T cell proliferation assay

PBMCs were labelled with Carboxyfluorescein succinimidyl ester (CFSE, ThermoFisher Scientific, Massachusetts, United States, #Cat: C34554) according to the manufacturer’s protocol and seeded into 96-well round-bottom plates (2x10^5^ cells per well) in RPMI 1640 medium (ThermoFisher Scientific, Massachusetts, United States, #Cat: 72400112) supplemented with 10% foetal bovine serum (FBS, ThermoFisher Scientific, Massachusetts, United States, #Cat: A5669701), 25 mM HEPES, 2 mM L-glutamine, and 1% Penicillin-Streptomycin (ThermoFisher Scientific, Massachusetts, United States, #Cat: 15140122). CFSE-labelled PBMCs were stimulated with either pokeweed mitogen (PWD, 2.5 μg/Ml, Sigma, Massachusetts, United States, #Cat: L8777-5MG) or Dynabeads Human T-activator CD3/CD28 (0.5 μL/well, ThermoFisher Scientific, Massachusetts, United States, #Cat: 11131D) for three days. Supernatants were collected, aliquoted, and stored at -80°C until use.

### 2.7 Activation of monocytes by immunomodulators

PBMCs were seeded into 96-well flat-bottom plates (based on 2x10^4^ monocytes per well) in RPMI 1640 medium supplemented (ThermoFisher Scientific, Massachusetts, United States, #Cat: 72400112) supplemented with 10% foetal bovine serum (FBS, ThermoFisher Scientific, Massachusetts, United States, #Cat: A5669701), 25 mM HEPES, 2 mM L-glutamine, and 1% Penicillin-Streptomycin (ThermoFisher Scientific, Massachusetts, United States, #Cat: 15140122). Cells were stimulated with lipopolysaccharide (LPS, Sigma, Massachusetts, United States, #Cat: L2630-10MG) to simulate Gram-negative bacterial infection, R848 (Resiquimod, InvivoGen, California, United States, #Cat: tlrl-r848-1) as a TLR7/8 agonist to simulate viral infection, or beta-glucan (β-glucan, InvivoGen, California, United States, #Cat: tlrl-wgp) to simulate fungal infection for 24 hours (early response) and seven days (late response). After stimulation, supernatants were collected, aliquoted, and stored at -80°C.

### 2.8 Soluble immune checkpoint and cytokine quantification

Soluble immune checkpoints and cytokine concentrations in plasma samples and supernatants were measured using the LEGENDplex Human Immune Checkpoint Panel 1 (12-plex: sCD25, 4-1BB, sCD27, sCD86, sCTLA-4, sPD-L1, sPD-L2, sPD-1, sTim-3, sLAG-3, Gal-9 and TGF-β; Biolegend, California, United States, #Cat: 740867) and LEGENDplex Human Essential Immune Response Panel (13-plex: IL-1β, IL-2, IL-4, IFNγ, TNF-α, MCP-1, CXCL10, IL-6, IL-8, IL-10, IL-12p70, IL-17A, and Free Active TGF-β1; Biolegend, California, United States, #Cat: 740930), according to the manufacturer’s instructions. Samples were acquired on a FACSCalibur flow cytometer (BD Biosciences, New Jersey, United States), and data were processed using LEGENDplex software (v.8, Biolegend).

### 2.9 Statistics

Raw data from immune factors and COVID-19 history were normalised using the Z-score method. Hierarchical clustering was performed using R (v.4.0.2) with the *heatmap*, *geom_tile*, and *ggplot2* packages (v.1.16.0). The overall accuracy was calculated by dividing the number of correctly classified samples in the two-group analysis by the total number of samples. Principal Component Analysis (PCA) was conducted using the *prcomp*, *princomp* and *PCA* packages with k-nearest neighbours (kNN) and Euclidean clustering.

Group differences were assessed using Student’s t-test and Levene’s test, with statistical significance set at p<0.05. Receiver Operating Characteristic (ROC) curve analysis identified potential predictors, with optimal cut-offs determined by the Youden Index.

Due to the nature of the SPSS software, the binary logistic regression model was performed on a subset of cases that presented all the available variables, comprising 10 PAHUA patients and 11 non-PAHUA cases. The binary logistic regression model predicted PAHUA using nine variables with AUC/ROC>0.9 and statistically significant in univariate regression applying Wald forward stepwise regression (13, 14). The final model combined Gal-9 and sTim-3, expressed as: Score = 0.00178 x Gal-9 levels + 0.03321 x sTim3 levels. 

To further evaluate the predictive validity and generalisability of the binary logistic regression model, internal validation was conducted using R (version 4.3.3). A 10-fold cross-validation was carried out with the *caret* package, assessing predictive performance via the area under the curve (AUC), which yielded a mean AUC of 1. Sensitivity and specificity were both estimated at 0.9.

Prior to validation, variable selection was performed using the *MASS* package, applying a forward stepwise logistic regression approach based on the Wald statistic. In addition, a non-parametric bootstrap procedure comprising 10,000 iterations was implemented with the *boot* package to estimate the stability and 95% confidence intervals of the AUC, resulting in a mean AUC of 0.999.

Receiver operating characteristic (ROC) curves and AUC values were calculated using the *pROC* package. The *dplyr* and *readxl* packages were employed for data pre-processing and importation, respectively.

Statistical analyses were performed with SPSS v.23 and GraphPad Prism 8.0 and R software.

## 3 Results

### 3.1 Clinical and epidemiological profiles of PAHUA patients

Between May 2022 and January 2023, 43 children were recruited from the Paediatric University Hospital La Paz. A discovery cohort of 29 participants included 21 children with acute hepatitis and eight healthy paediatric volunteers (HVs). Of these, 17 met PAHUA criteria per ECDC/WHO guidelines, while 4 were diagnosed with autoimmune hepatitis (AIH), which served as an internal control (**Figure 1**).

Most PAHUA patients presented with gastrointestinal symptoms such as vomiting, diarrhoea, and abdominal discomfort, occasionally accompanied by hepatomegaly or splenomegaly. These symptoms progressed to jaundice, with fever notably absent (**Supplementary Table S5**). Hepatitis viruses A–E were excluded, and no shared environmental exposures were identified.

The cohort’s median age was 7.2 years (range: 1–15), with gender distribution balanced (52% male) (**Table 1**). PAHUA patients exhibited significantly elevated bilirubin and liver enzyme levels (AST, ALT, and GGT) compared to HVs (**Table 1**). Anaemia, coagulopathy, abnormal INR and prothrombin activity were common among PAHUA cases. Ten PAHUA and one AIH patient developed paediatric acute liver failure (pALF); one required liver transplantation, and two succumbed to the illness. Treatments included plasmapheresis (24%), steroids (12%), intravenous immunoglobulin (IVIG) (6%), and N-acetylcysteine (6%).

**Table 1:** Demographic and clinical characteristics of discovery cohort.

Variable	PAHUA (N=17)	Autoimmune (N=4)	HVs (N=8)	*p*-value (PAHUA *vs*. HVs)	*p*-value (PAHUA *vs*. AIH)
Median age (range) — yr	2.9 (1–14)	13.6 (8–15)	10.9 (1-14)	ns	*
Sex assigned at birth—no. (%)					
Female	5 (29)	4 (100)	5 (63)	ns	*
Male	12 (71)	0 (0)	3 (37)	ns	*
Median white-cell count (range) — per uL	6990 (3300–24000)	5620 (1780–6770)	8210 (4950-11840)	ns	ns
Median serum bilirubin level (range) — mg/dl	4.03 (0.43–17.52)	1.95 (0.92–4.69)	0.77 (0.30–1.2)	*	ns
Median AST level (range) — IU/liter	3400 (627–16477)	343 (75–591)	33 (30–44)	****	***
Median ALT level (range) — IU/liter	3420 (490–10873)	258 (79–1108)	19 (17–35)	**	*
Median GGT (range) — IU/liter	92 (34–525)	61 (33–115)	14 (13–17)	***	ns
Median LDH (range) — IU/liter	989 (340-12691)	245 (172-307)	–	–	***
Median Ammonium (range) – ug/dL	125 (15-345)	100.3 (70-105)	–	–	ns
Median INR (range)	1.40 (1-5.8)	1.35 (1.1-2.1)	1.0 (1-1.1)	**	ns
Median Fibrinogen (range) — mg/dl	162 (129-552)	162 (85-251)	271 (234-271)	ns	0.054
Median Prothrombin activity (range) — percentage	54 (9-103)	60 (32-86)	103 (78-110)	**	ns
Median serum albumin (range) —g/dl	3.8 (2.6-4.7)	3.5 (2.3-4.8)	4.4 (4.4-4.4)	**	ns
Clinical progression					
Anaemia	14 (82)	2 (50)	0 (0)	***	ns
Coagulopathy	15 (88)	2 (50)	0 (0)	***	ns
Paediatric Acute Liver Failure (PALF)	10 (59)	1 (25)	–	–	ns
Liver transplant	0 (0)	1 (25)	–	–	ns
*Exitus*	1 (6)	1 (25)	–	–	ns

LDH, Lactate Dehydrogenase; INR, International normalised ratio; IVIG, Intravenous immunoglobulin; PAHUA, Paediatric acute hepatitis of unknown aetiology; HV, Healthy paediatric volunteer. *p-value<0.05; **p-value<0.01; ***p-value<0.001; ****p-value<0.0001.

Adenovirus was detected in 17.6% of PAHUA patients, with two cases co-infected with influenza A (**Supplementary Table S6**). Additionally, 41% tested positive for other infections, including norovirus, cytomegalovirus (CMV), and herpesvirus. SARS-CoV-2 IgG antibodies against the spike protein were found in 88.2% of PAHUA patients and 100% of AIH and HVs, indicating prior infection rather than vaccination (**Supplementary Table S6**). Nucleocapsid protein IgG analysis revealed that 11.8% of PAHUA patients lacked evidence of prior SARS-CoV-2 infection, supporting hypotheses of immune-mediated liver inflammation potentially linked to the spike protein acting as a superantigen (11).

### 3.2 Differential clustering of PAHUA, AIH and HV cases based on immunological data

To investigate immune profiles in PAHUA and non-PAHUA (AIH and HV) groups, immunophenotyping of whole blood was performed using spectral flow cytometry, alongside *ex vivo* immune response assessments, T cell proliferative capacity, and quantification of cytokine and soluble immune checkpoints (ICs) levels (**Supplementary Figure S1**).

**Figure 2:**
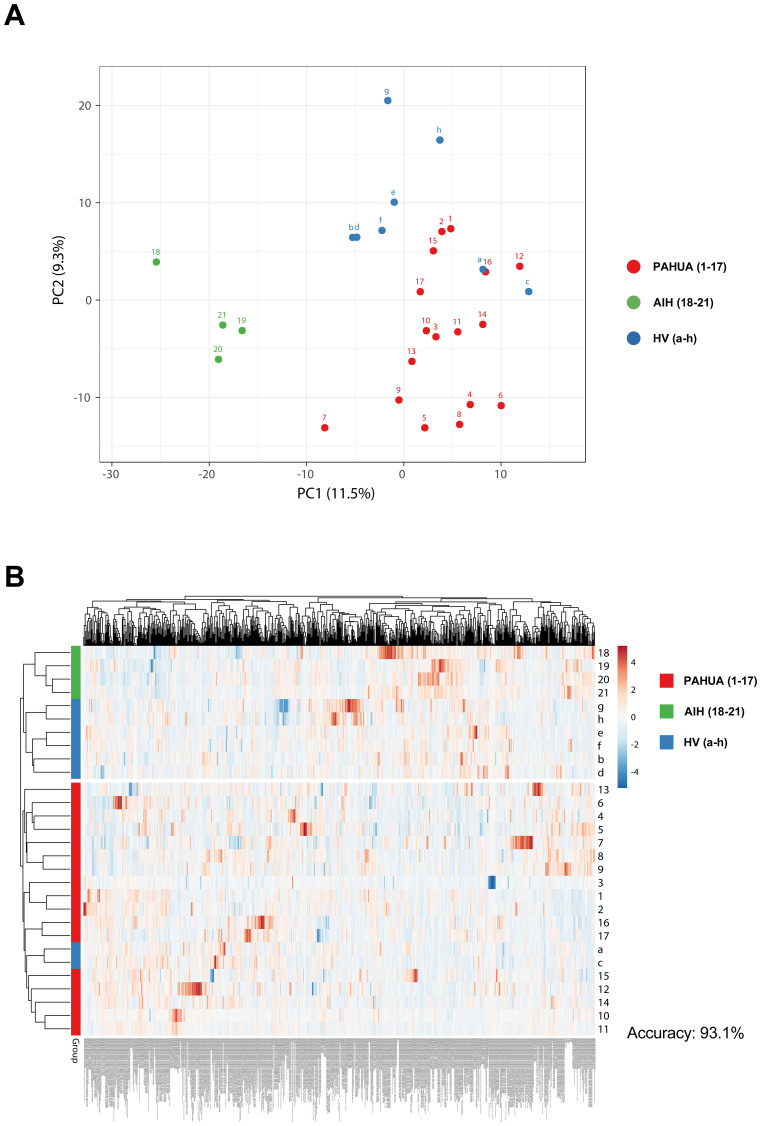
PAHUA, AIH, and HV cases exhibit a differential clustering pattern in accordance with all their clinical and immunological data. **(A)** Principal component analysis (PCA) including all immunological data from PAHUA (1-17; red dots), AIH (18-21; green dots), and HV cases (a-h; blue dots). **(B)** Two-group heatmap including all immunological data from PAHUA (1-17; red squares), AIH (18-21; green squares), and HV cases (a-h; blue squares).

Combining all immunological data, principal component analysis (PCA) revealed distinct clustering patterns among PAHUA (red: 1-17), AIH (green: 18-21), and HV (blue: a-h) groups (**Figure 2A**). AIH cases were most distinct, separating undoubtedly from PAHUA and HV groups. Two-group heatmap analysis using Euclidean clustering and correlation grouping achieved 93.1% overall accuracy, segregating non-PAHUA cases (top cluster) from PAHUA patients (bottom cluster). Two HVs (a and c cases) grouped with PAHUA patients, likely due to prior infection with SARS-CoV-2 (a) and HIV exposure during pregnancy (c) (**Figure 2B**).

Analyses focusing on specific datasets showed varying accuracies. Whole blood immunophenotyping, analysing 529 immune cell populations, achieved 86.2% accuracy in the two-group heatmap analysis, though group separation was less distinct in the PCA (**Supplementary Figure S2**). In this analysis, the top cluster included most of the non-PAHUA patients, with two PAHUA patients (7 and 13). As observed in the previous heatmap (**Figure 2B**), which incorporated all immunological data, two HVs (a and c) were again grouped with the PAHUA patients in the bottom cluster. In contrast, PCA and heatmap analyses based on T cell proliferation and monocyte phenotyping following various stimulations yielded worst results, achieving an overall accuracy of 65.5% (**Supplementary Figure S3**). The two-group heatmap revealed that most non-PAHUA cases clustered in the top group, which also included seven PAHUA patients (1–6 and 15). The bottom cluster composed of the remaining PAHUA patients and three HVs (a, b and c).

Given the potential of soluble molecules as biomarkers and therapeutic targets (15–17), we assessed their capacity to classify the three groups. ICs achieved 79.3% accuracy, outperforming cytokines (75.9%), but neither matched the full dataset’s accuracy (**Supplementary Figures S4, S5**). Nevertheless, the showed promise in distinguishing PAHUA from other groups and warrant further investigation as biomarkers.

### 3.3 Volcano plot and variable enrichment analysis identify key prognostic variables for PAHUA

A volcano plot analysis identified differentially expressed variables between PAHUA, AIH, and HV groups (**Supplementary Figure S6**). Using a p-value threshold of 0.01 and a fold-change of 2, we found 15 upregulated and eight downregulated variables in PAHUA *vs*. HV; 28 upregulated and 23 downregulated in PAHUA *vs*. AIH; and 17 upregulated and 18 downregulated in AIH *vs*. HV (**Supplementary Figure S6A**).

Immunophenotyping variables were most frequently highlighted and were effective in clustering the groups (**Supplementary Figure S7**). Notably, Gal-9 and sTim-3 were commonly upregulated in PAHUA compared to both HV and AIH, marking them as key biomarkers (**Supplementary Figure S6B** and **Supplementary Table S7**).

### 3.4 Soluble immune checkpoints score predict PAHUA condition

**Figure 3:**
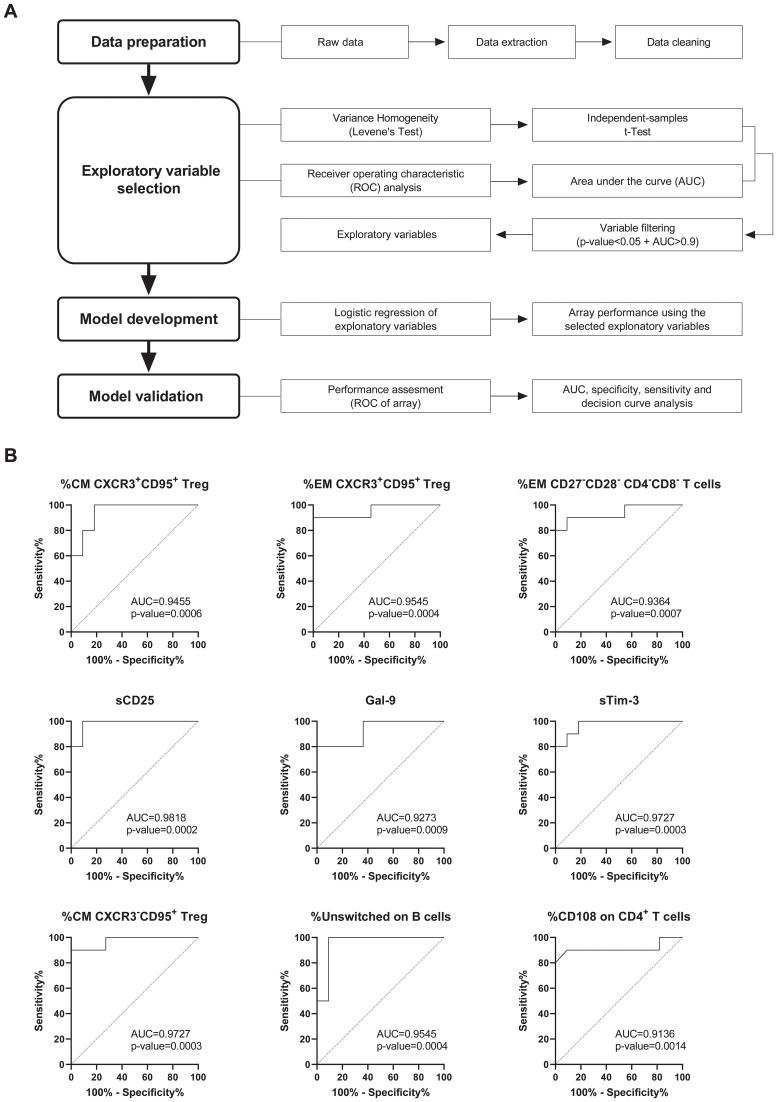
Development of a prognostic tool for PAHUA based on bioinformatics analysis. **(A)** Workflow of the bioinformatics approach employed to identify the exploratory variables that differentiate PAHUA patients from non-PAHUA cases (AIH and HVs). A total of 790 variables, including immune cell populations in whole blood and data from *ex vivo* experiments, were evaluated using AUC/ROC and univariate logistic regression. Variables with an AUC>0.90 and a statistical significance (p-value<0.05) were incorporated into a binary logistic regression model to perform an array score, which was subsequently validated. **(B)** ROC curves analysis of the nine exploratory variables for PAHUA patients identified after the AUC/ROC and univariate logistic regression analysis. Calculated AUCs of the ROC curves and p-values are indicated for each exploratory variable.

To develop a reliable prognostic tool for PAHUA, we assessed 790 variables, including COVID-19 history (3), immune cell populations (529) and *ex vivo* data (258), using AUC/ROC and univariate logistic regression. Variables with AUCs>0.90 and statistically significant results by univariate logistic regression (p<0.05) were incorporated into a binary logistic regression model (**Figure 3A**).

Nine variables were identified: %CM (central memory) CXCR3^+^CD95^+^ Treg (T regulatory), %EM (effector memory) CXCR3^+^CD95^+^ Treg, %EM CD27^-^CD28^-^ CD4^-^CD8^-^ T cells, sCD25, Gal-9, sTim-3, %CM CXCR3^-^CD95^+^ Treg, %Unswitched on B cells, and %CD108 on CD4^+^ T cells. Each variable demonstrated strong discrete predictive capacity, with AUCs ranging from 0.9136 (%CD108 on CD4^+^ T cells) to 0.9818 (sCD25) (**Figure 3B**). Optimal cut-off values were determined using the Youden Index (**Supplementary Figure S8**).

**Figure 4:**
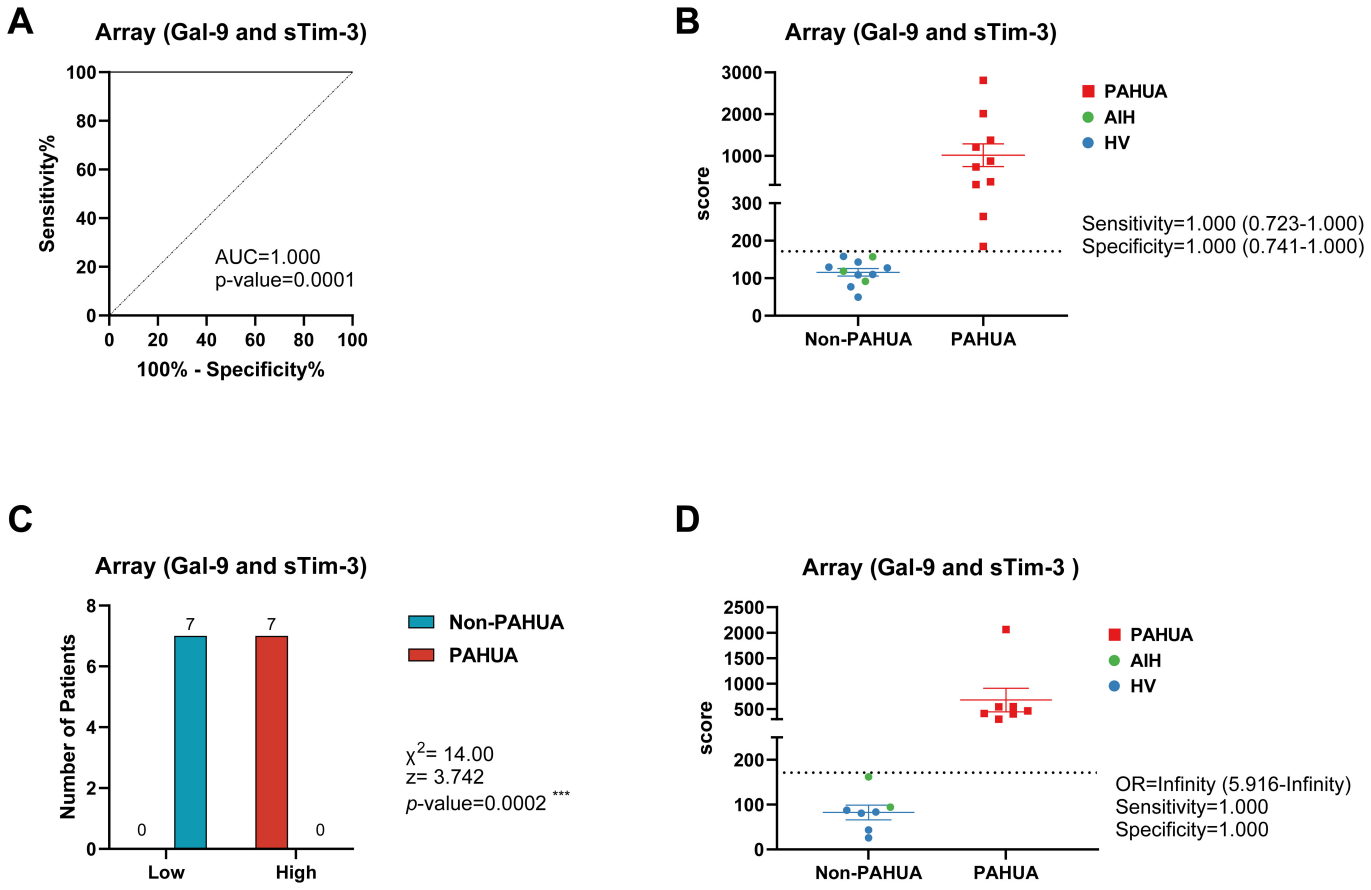
Score from logistic mathematical model predicted the PAHUA condition in a validation cohort. **(A)** ROC curve analysis for PAHUA prediction in discovery cohort (PAHUA, n=17; non-PAHUA, n=12) of the score obtained from binary logistic regression model including the both soluble immune checkpoints Gal-9 and sTim-3. **(B)** Estimated score of discovery cohort according to their plasmatic Gal-9 and sTim-3 levels. Dashed line indicates the optimal Youden cut-off for PAHUA prediction. Sensitivity and specificity of this cut-off is shown. **(C)** Validation cohort (PAHUA, n=7; non-PAHUA, n=7) were classified according to their plasmatic Gal-9 and sTim-3 levels and the regression model score (low and high subgroups by using optimal Youden cut-off). Patients number distribution and chi-square test statistics are shown. **(D)** Estimated score of validation cohort according to their plasmatic Gal-9 and sTim-3 levels. Dashed line indicates the optimal Youden cut-off for PAHUA prediction. Odds ratio (OR), sensitivity and specificity of this cut-off is shown. X^2^, Chi-square; z, z-statistic.

The nine variables were incorporated into a forward stepwise regression model (Wald test). After three steps, the final optimised model included the combination of two ICs, Gal-9 and sTim-3, both of which are easily quantifiable in clinical settings. This resulted in a highly effective predictive tool, achieving an AUC of 1.000 (95% CI 1.000–1.000) in ROC analysis with a statistically significant p-value of 0.0001 (**Figure 4A**). The optimal cut-off score, determined using the Youden Index, provided maximum sensitivity (1.000; 95% CI 0.723–1.000) and specificity (1.000; 95% CI 0.741–1.000), effectively distinguishing PAHUA cases from non-PAHUA cases (AIH and HV) (**Figure 4B**). It is important to note that into the non-PAHUA individuals, the AIH patients (green dots) were separated totally from the PAHUA patients.

To validate the model, Gal-9 and sTim-3 levels were quantified in the validation cohort (PAHUA, n=7 and non-PAHUA, n=7). The Chi-squared test confirmed significant differences between high and low scoring groups (χ²=14.00; z=3.742; p=0.0002), with perfect sensitivity and specificity in distinguishing PAHUA cases (**Figure 4C**). This finding underscores the discriminatory power of the predictive model in distinguishing between patient groups.

Furthermore, the predictive score demonstrated perfect specificity (1.000; 95% CI 0.646–1.000) and sensitivity (1.000; 95% CI 0.646–1.000) in differentiating PAHUA from non-PAHUA cases (**Figure 4D**).

## 4 Discussion

This study provides critical insights into the clinical and immunological characteristics of paediatric acute hepatitis of unknown aetiology (PAHUA), offering a detailed profile that distinguishes it from healthy paediatric individuals (HV) and autoimmune hepatitis (AIH) patients. These findings contribute significantly to understanding PAHUA’s pathophysiology and lay the groundwork for improved diagnostic and therapeutic strategies.

Clinically, PAHUA patients predominantly presented with gastrointestinal symptoms, such as vomiting, diarrhoea, and abdominal discomfort, preceding jaundice. Hepatomegaly and, occasionally, splenomegaly were observed in agreement with other studies (18, 19), while fever was notably absent, contrasting with typical viral infections (4, 20). Exclusion of hepatitis viruses A–E and the absence of shared environmental exposures further complicate the search for a causative agent (1–3, 6). The progression to paediatric acute liver failure (pALF), characterised by coagulopathy and elevated liver enzymes, underscores the severity of the condition as it has been reported in other cohorts (21, 22). Remarkably, 90% of our PAHUA cohort recovered from pALF, surpassing the 25% native liver survival reported in other studies (23, 24).

Our findings challenge the notion of adenovirus as the primary causative agent. Only 17.6% of PAHUA patients tested positive for adenovirus, with many cases exhibiting co-infections, suggesting that adenovirus may play a secondary role. A previous study involving 274 patients identified a significant association between adenoviral viremia and acute paediatric hepatitis of unknown aetiology, reporting a 68% positivity rate for the virus (12). In contrast, the robust immune response to SARS-CoV-2, evidenced by the presence of Spike protein IgG antibodies in 88.2% of PAHUA patients, lends credence to the hypothesis that SARS-CoV-2 may function as a superantigen (25–27), precipitating immune-mediated liver inflammation in predisposed individuals (11). This hypothesis is further supported by a growing body of evidence underscoring SARS-CoV-2’s hepatotropism (28–30).

Immune profiling revealed significant dysregulation in PAHUA patients compared to AIH and HV groups. Immunophenotyping and *ex vivo* assays demonstrated the presence of distinct immune signatures in PAHUA, accompanied by elevated serum bilirubin and liver enzymes. Clustering analyses effectively distinguished PAHUA cases, with overlapping profiles in two HVs attributed to prior exposure to viruses (SARS-CoV-2 and HIV). Thus, the integration of immunological data for PAHUA classification demonstrated its utility for the diagnosis and management of this condition.

Nine variables, including three soluble immune checkpoints (sCD25, Gal-9, and sTim-3) and specific immune cell populations, demonstrated strong predictive power for diagnosing PAHUA, each achieving an AUC>0.90. A predictive score combining Gal-9 and sTim-3 achieved perfect sensitivity and specificity in both discovery and validation cohorts, highlighting their potential as minimally invasive biomarkers. Although PAHUA condition is rare, which explains our models sample size, we recognise that working with small cohort can reduce statistical power and increase the risk of overfitting in complex models, as we have used in this study. To help address this, we performed a 10-fold cross-validation of the binary logistic regression model, which yielded a mean AUC of 1.000 across all folds, indicating excellent predictive performance and minimal risk of overfitting. Moreover, we used bootstrap resampling (10000 iterations) to test the reliability of our binary logistic regression model, indicating it performs reliably (AUC=0.999). The combination of bootstrapping and cross-validation enhance the confidence in the reliability and generalisability of the model despite the limited cohort size.

While these findings are highly promising and despite the Gal-9/sTim-3 model demonstrating perfect accuracy in both discovery and validation cohorts, the limited size of the validation group restrict the generalisability of the findings. This constraint must be considered when interpreting the clinical applicability of the model, and underscores the necessity for larger multicentre studies to refine threshold values and confirm performance in diverse populations.

Note that, although further exploration in a larger cohort of these biomarkers across a broader range of liver conditions could clarify their specificity and utility in differential diagnosis, the score incorporating Gal-9 and sTim-3 levels was also able to discriminate between PAHUA and AIH patients in both discovery and validation cohorts. This finding underscores the potential of this score in distinguishing patients with similar clinical presentations but with immunological discrepancies.

These soluble ICs can be quantified using routine immunoassays, enabling swift and accurate diagnosis without invasive procedures like liver biopsies. This enhances diagnostic precision and clinical decision-making in this emerging paediatric liver disease. Integrating Gal-9 and sTim-3 into routine diagnostic workflows has the potential to transform PAHUA diagnosis, enabling earlier intervention and significantly improving patient outcomes.

This study marks a pivotal advancement in our understanding of PAHUA, introducing powerful diagnostic tools that lay the foundation for future research into its aetiology and the development of targeted therapeutic strategies.

## Data Availability

The raw data supporting the conclusions of this article will be made available by the authors, without undue reservation.
