# Supplementary material for: Comprehensive immune profiling and predictive modelling of paediatric acute hepatitis of unknown aetiology from a Spanish cohort
Source: Front Immunol. 2025 Jun 24;16:1599982. doi: 10.3389/fimmu.2025.1599982 (PMC12235265; doi:10.3389/fimmu.2025.1599982)
Supplement: Supplementary file 1 [file Table1.docx]

Supplementary Material

# Supplementary Figures and Tables

## Supplementary Figures


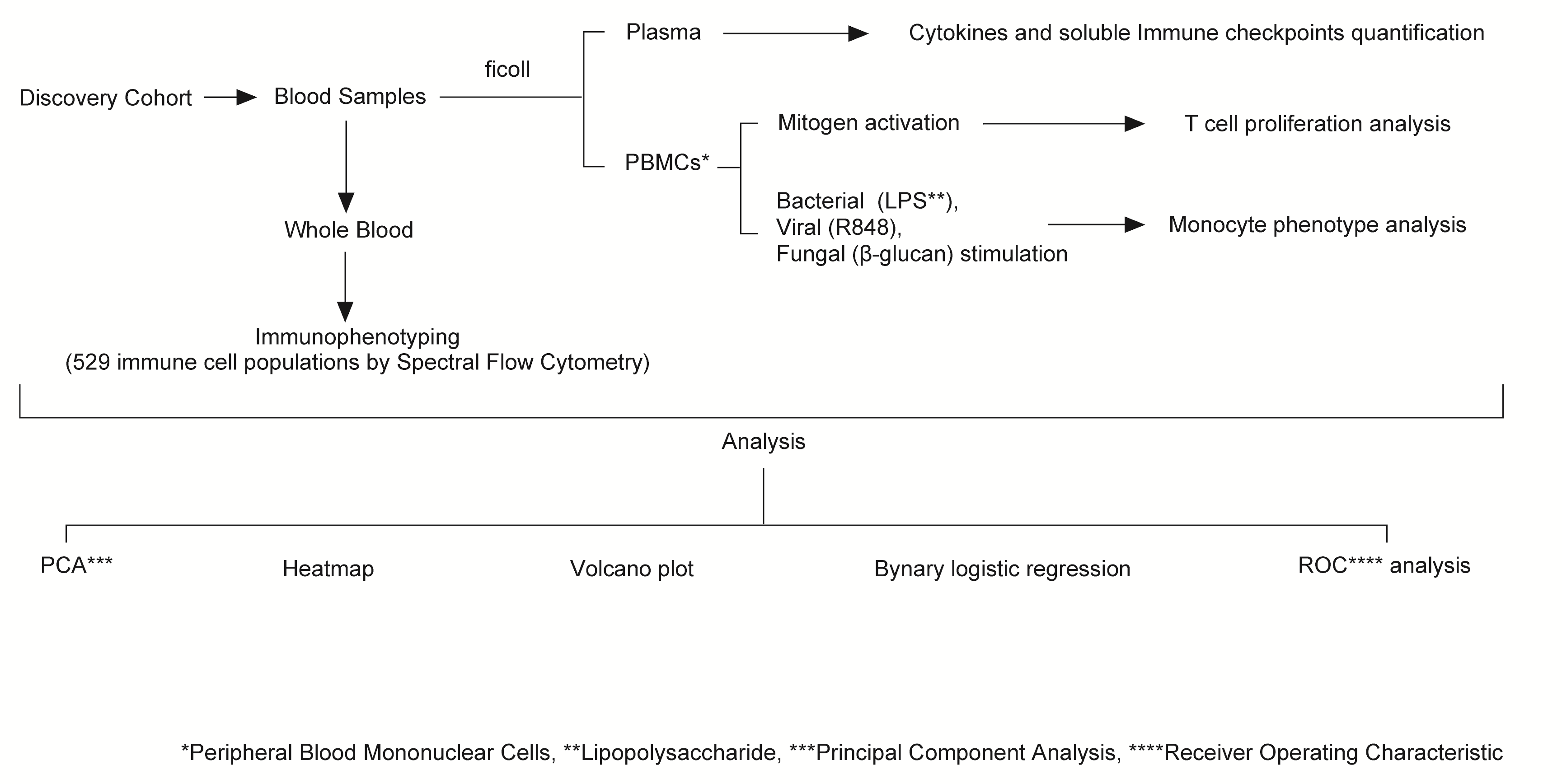


**Supplementary Figure 1.** **Comprehensive immune profiling in PAHUA and control groups (AIH and HV).** Workflow of the immunological analysis employed to provide insights into the immune cell populations on whole blood and the immune function and response profiles across the groups. Whole blood immunophenotyping was conducted using spectral flow cytometry to characterize immune cell populations in detail. *Ex vivo* immune response assessments included reactions to bacterial, viral, and fungal pathogens, evaluating T cell proliferation, cytokine levels, and soluble immune checkpoints.


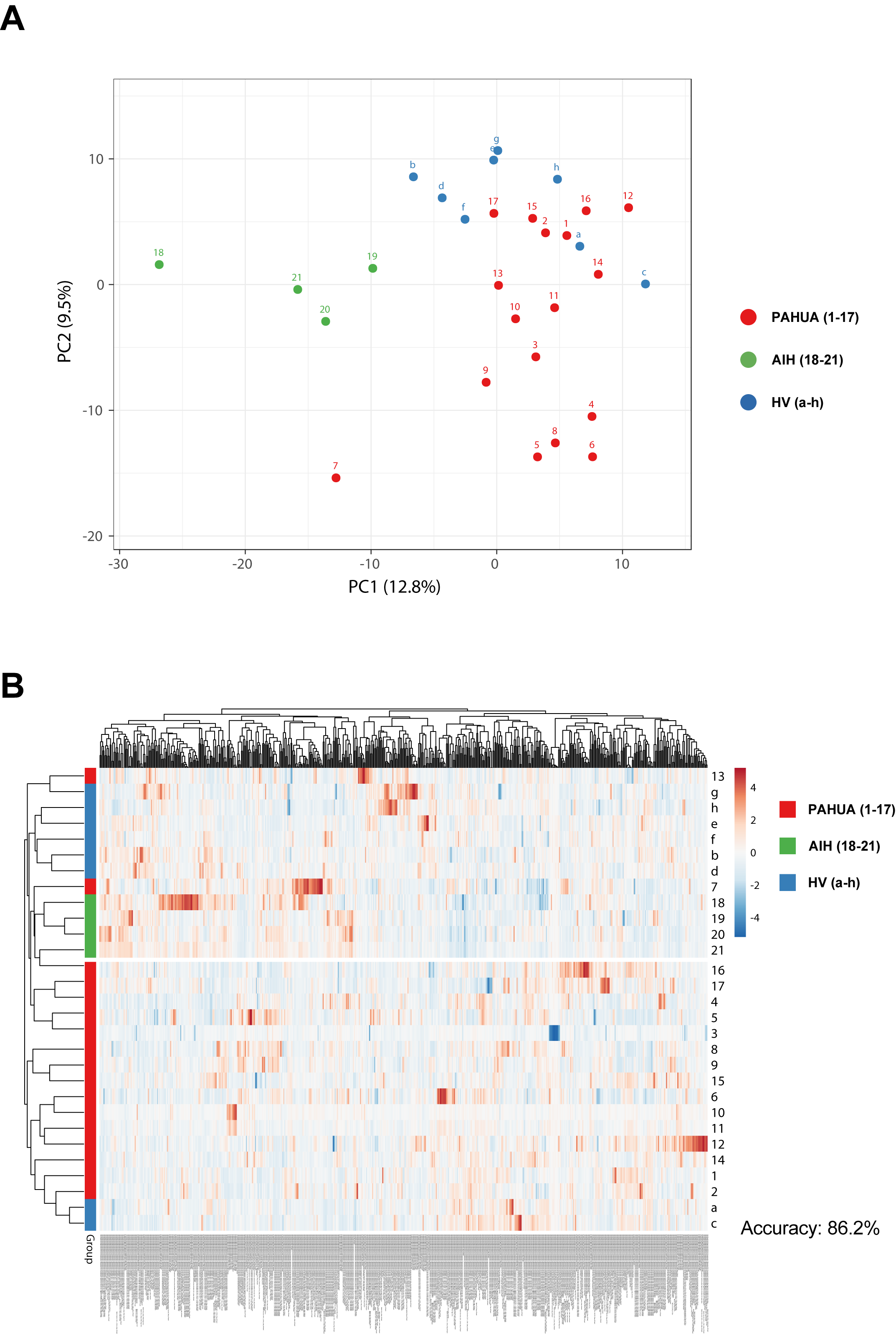


**Supplementary Figure 2.** **Clustering of PAHUA, AIH, and HV cases in accordance with their immune cell populations on whole blood. (A)** Principal component analysis (PCA) including immune cell populations on whole blood from PAHUA (1-17; red dots), AIH (18-21; green dots) and HV cases (a-h; blue dots). (**B**) Two-group heatmap including the immune cell populations on whole blood from PAHUA (1-17; red squares), AIH (18-21; green squares) and HV cases (a-h; blue squares).


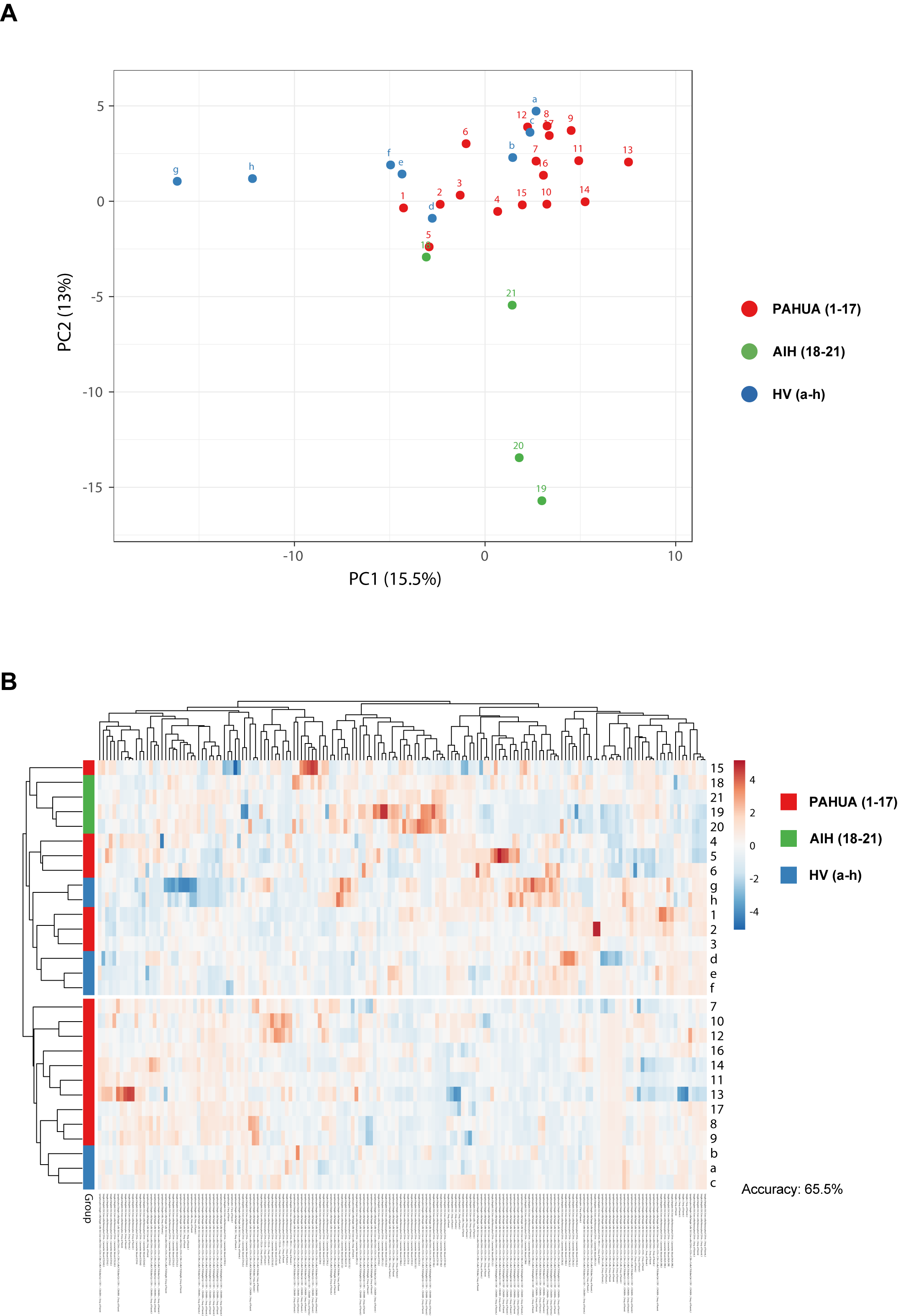


**Supplementary Figure 3. Clustering of PAHUA, AIH, and HV cases in accordance with their T cell proliferation and monocyte phenotyping following bacterial, viral and fungal pathogens stimulation. (A)** Principal component analysis (PCA) including the T cell proliferation and monocyte phenotyping following bacterial, viral and fungal pathogens stimulation from PAHUA (1-17; red dots), AIH (18-21; green dots) and HV cases (a-h; blue dots). (**B**) Two-group heatmap including the T cell proliferation and monocyte phenotyping following bacterial, viral and fungal pathogens stimulation from PAHUA (1-17; red squares), AIH (18-21; green squares) and HV cases (a-h; blue squares).


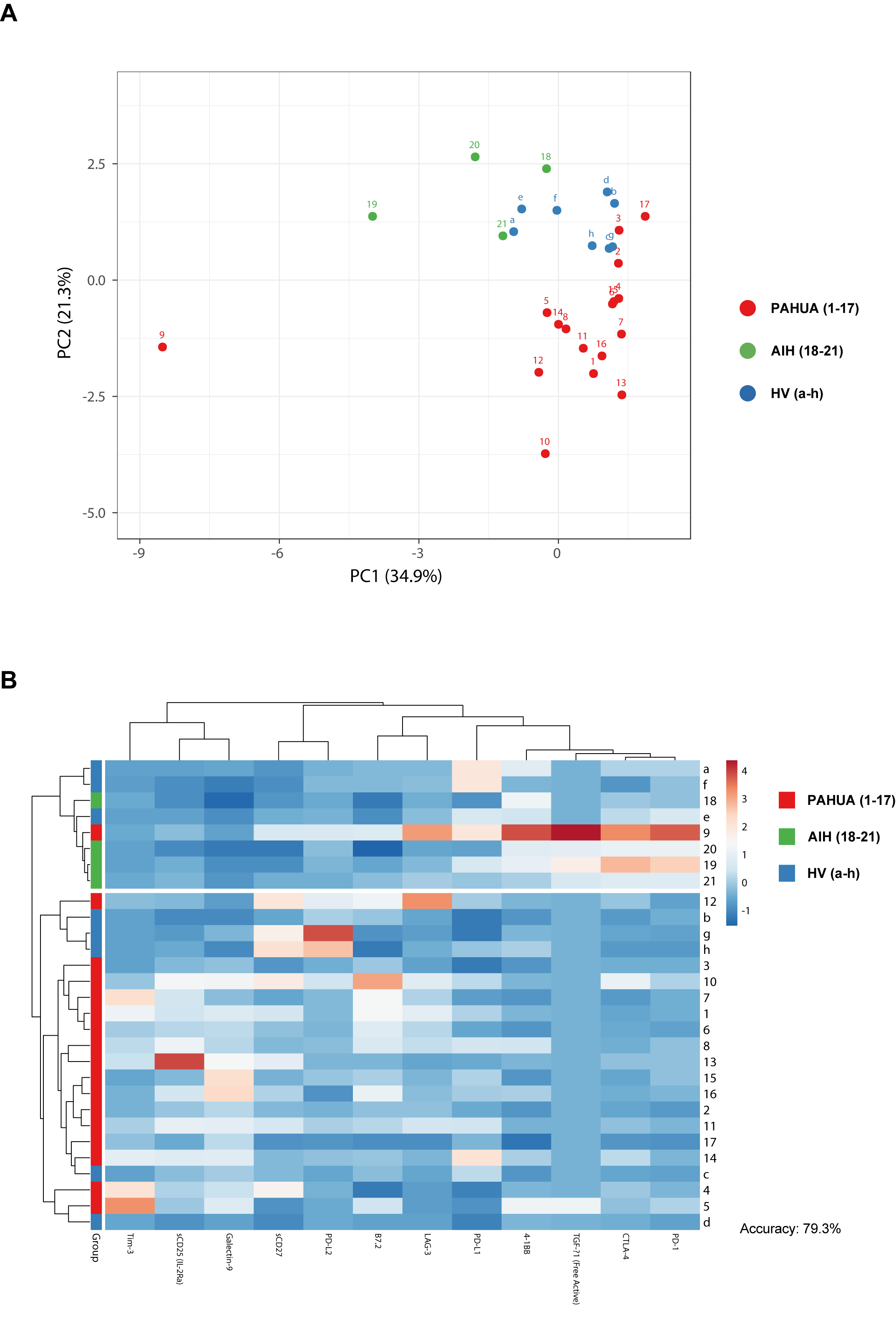


**Supplementary Figure 4. Clustering of PAHUA, AIH, and HV cases in accordance with their plasmatic immune checkpoints. (A)** Principal component analysis (PCA) including the plasmatic immune checkpoints from PAHUA (1-17; red dots), AIH (18-21; green dots) and HV cases (a-h; blue dots). (**B**) Two-group heatmap including the plasmatic immune checkpoints from PAHUA (1-17; red squares), AIH (18-21; green squares) and HV cases (a-h; blue squares).


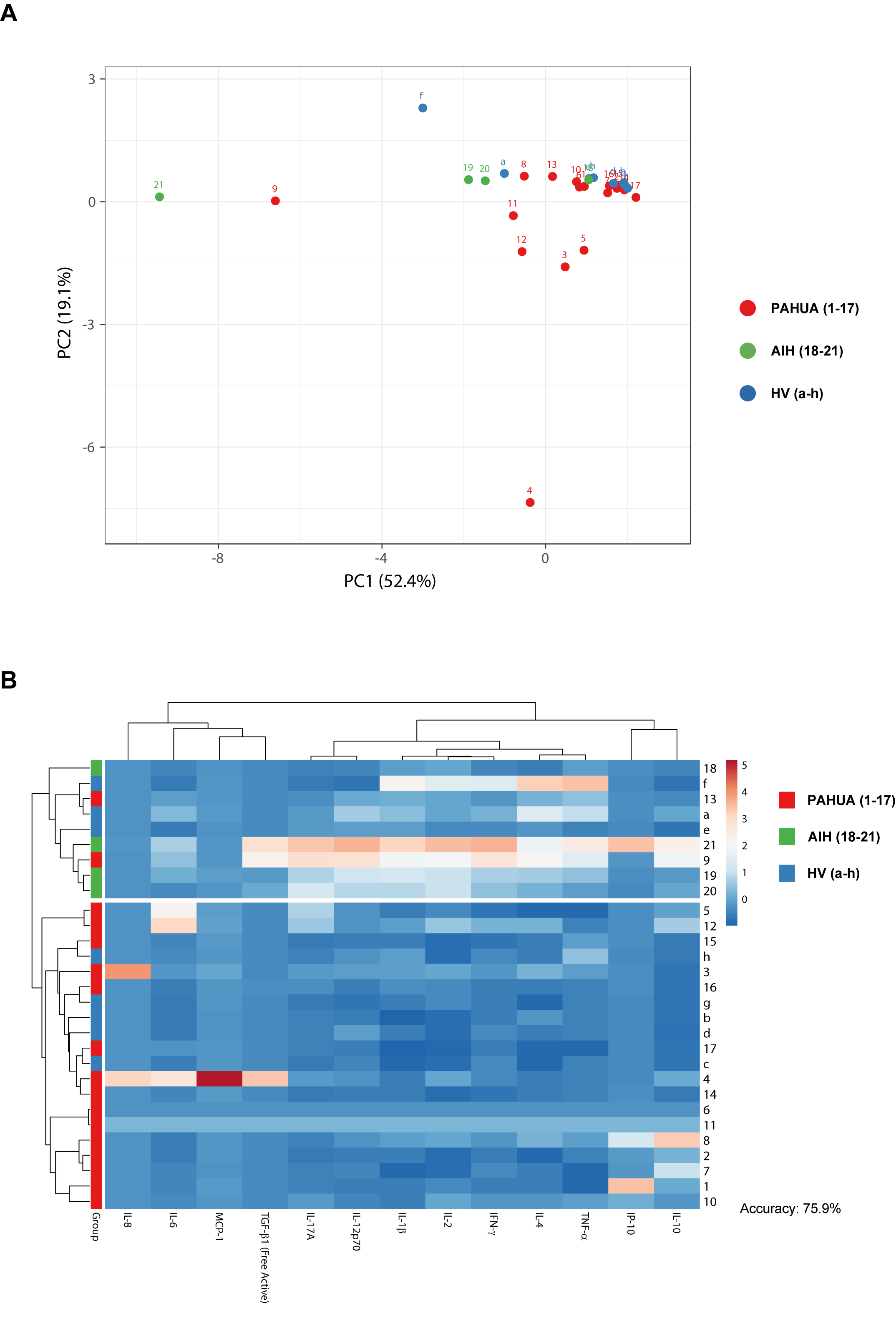


**Supplemetary Figure 5. Clustering of PAHUA, AIH, and HV cases in accordance with their plasmatic cytokines. (A)** Principal component analysis (PCA) including the plasmatic cytokines from PAHUA (1-17; red dots), AIH (18-21; green dots) and HV cases (a-h; blue dots). (**B**) Two-group heatmap including the plasmatic cytokines from PAHUA (1-17; red squares), AIH (18-21; green squares) and HV cases (a-h; blue squares).


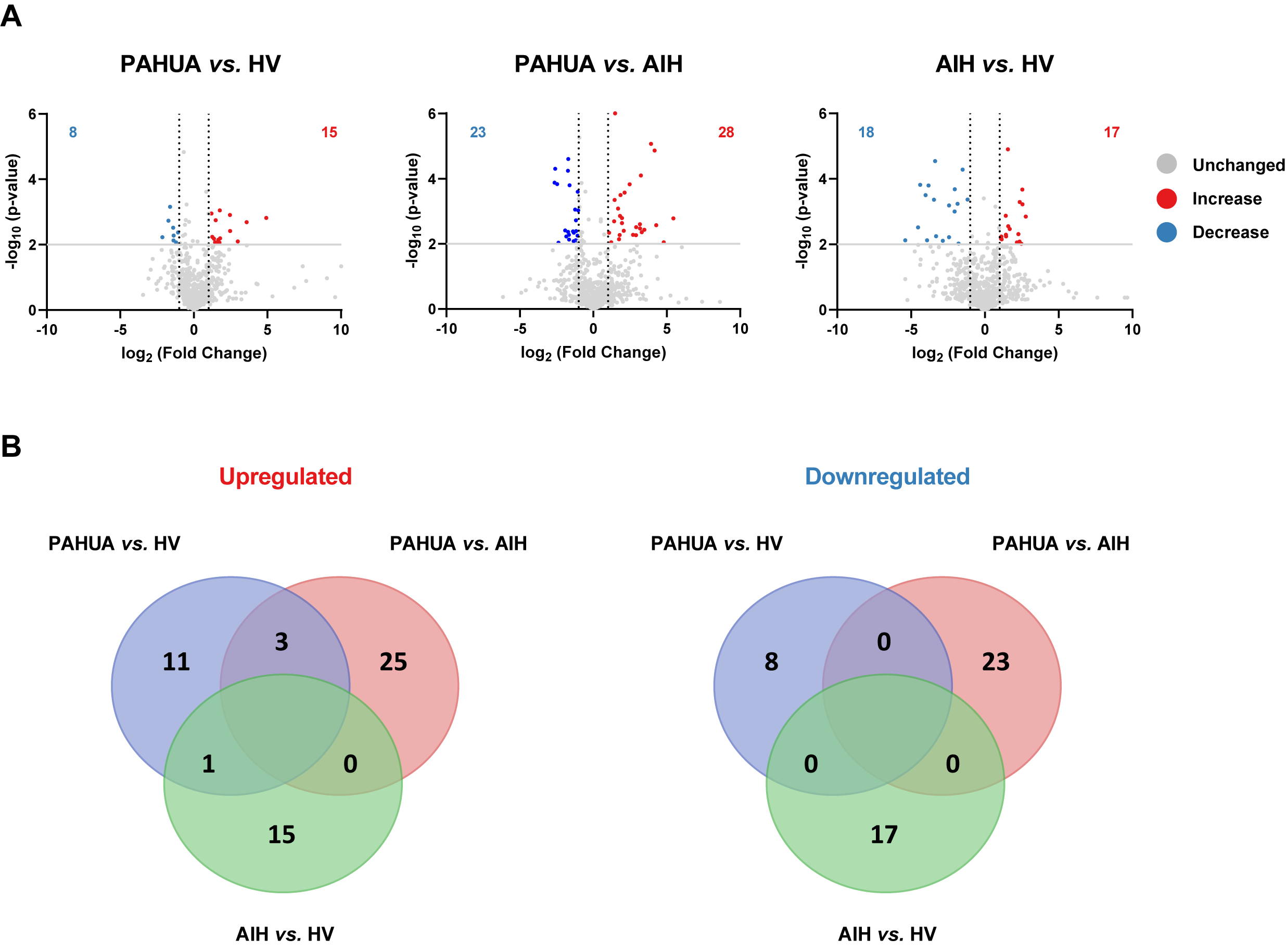


**Supplementary Figure 6. Comparative analysis of upregulated and downregulated variables in PAHUA, AIH and HV cases. (A)** Volcano plots show the upregulated (red dots) and downregulated (blue dots) variables in the PAHUA *vs.* HV (left) , PAHUA *vs.* AIH (middle) and AIH *vs.* HV comparisons (right) expressed as the logarithmic hazard on the x-axis versus p-values (expressed on an inverted logarithmic scale) on the y-axis for each clinical and immunological variable. **(B)** Venn diagram of differentially upregulated (left) and downregulated (right) variables and their overlapping for PAHUA, AIH and HV groups.


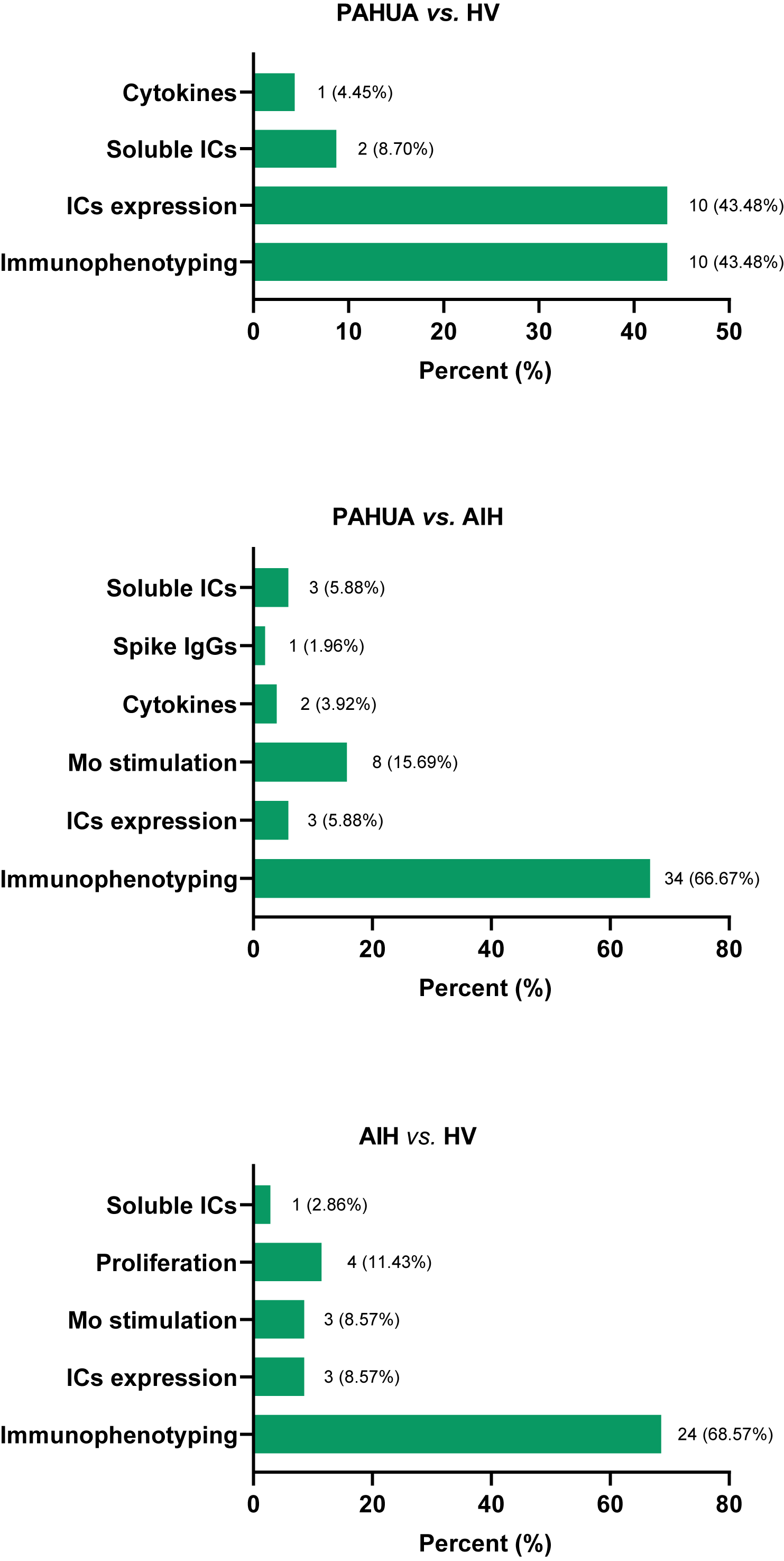


**Supplementary Figure 7. Enrichment analysis of upregulated and downregulated variables across the comparative analysis in PAHUA, AIH and HV cases.** The bar plots depict the percentage of the variable type in the PAHUA *vs.* HV (upper panel), PAHUA *vs.* AIH (center panel) and AIH *vs.* HV (lower panel) comparisons. ICs, immune checkpoints; Mo, monocyte.


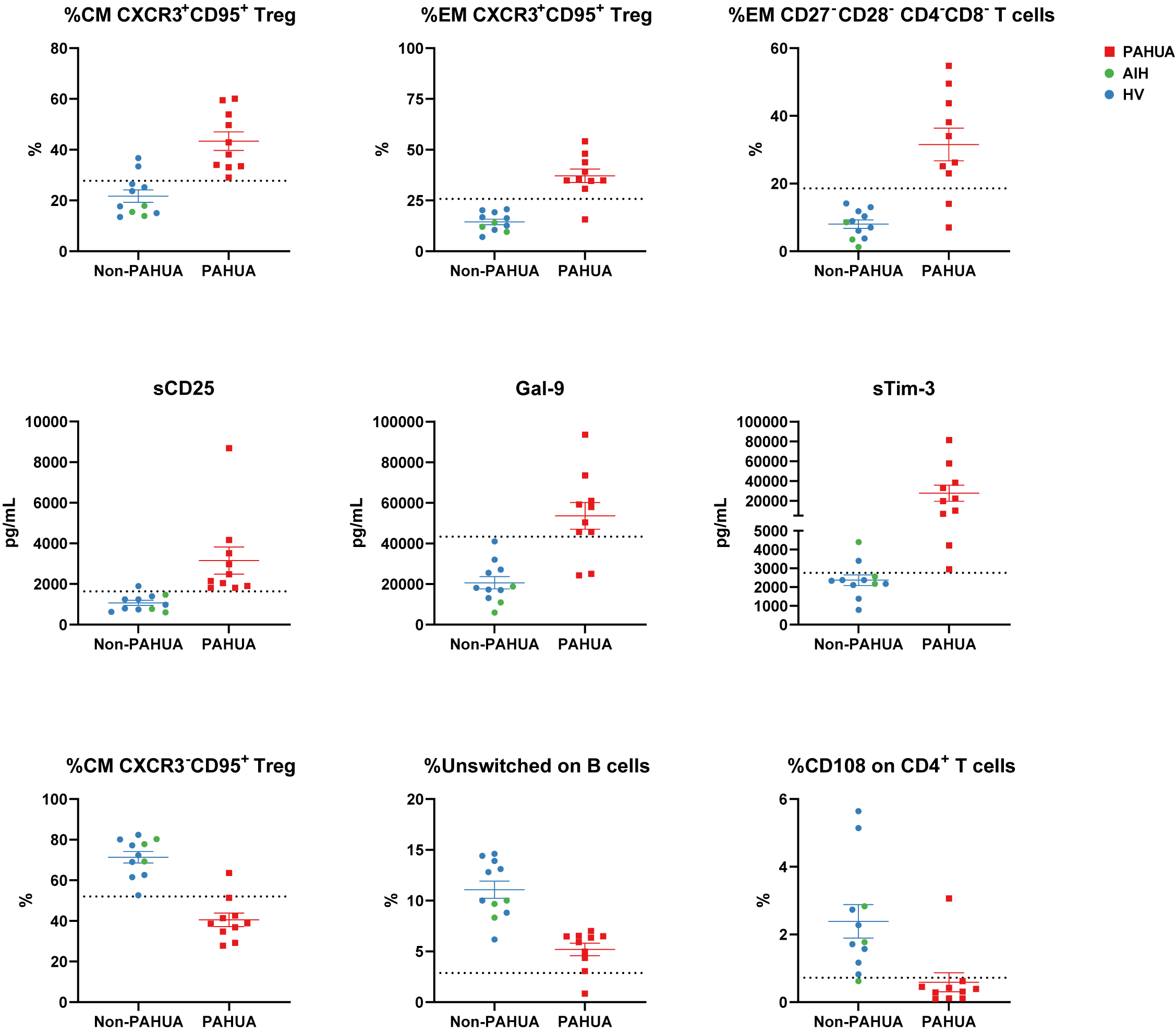


**Supplementary Figure 8. Classification performance of exploratory variables identified for PAHUA condition.** Estimated score of the discovery cohort according to the nine exploratory variables for the PAHUA condition (red squares) compared to control group (AIH, green dots; HV, blue dots) identified after the AUC/ROC and univariate logistic regression analysis.

## Suplementary Tables

**Supplementary Table 1. List of all fluorochrome-conjugated monoclonal antibodies for flow cytometry analysis of immune cell populations.**

| Marker | Fluorochrome | Source | Clone | Catalog number (RRID) |
| --- | --- | --- | --- | --- |
| CD45RA | BUV395 | BD | 5H9 | 740315 (AB_2740052) |
| CD16 | BUV496 | BD | 3G8 | 612944 (AB_2870224 |
| CD195 (CCR5) | BUV563 | BD | 2D7/CCR5 | 741401 (AB_2870893) |
| CD62L | BV615 | BD | SK11 | 751364 (AB_2875371) |
| CD11c | BUV661 | BD | B-ly6 | 612967 (AB_2870241) |
| CD56 | BUV737 | BD | NCAM16.2 | 612766 (AB_2813880) |
| CD8 | BUV805 | BD | SK1 | 612889 (AB_2833078) |
| IgD | BV480 | BD | IA6-2 | 566138 (AB_2739536) |
| IgG | BV7605 | BD | G18-145 | 563246 (AB_2738092) |
| CD185 (CXCR5) | BV750 | BD | RF8B2 | 747111 (AB_2871862) |
| CD141 | BB515 | BD | 1A4 | 566017 (AB_2739462) |
| CD127 | APC-R700 | BD | HIL-7R-M21 | 565185 (AB_2739099) |
| CD197 (CCR7) | BV421 | Biolegend | G043H7 | 353208 (AB_11203894) |
| CD3 | BV510 | Biolegend | OKT3 | 317332 (AB_2561943) |
| IgM | BV570 | Biolegend | MHM-88 | 314517 (AB_10913816) |
| CD28 | BV650 | Biolegend | CD28.2 | 302946 (AB_2616855) |
| CD196 (CCR6) | BV711 | Biolegend | G034E3 | 353436 (AB_2629608) |
| CD279 (PD-1) | BV785 | Biolegend | EH12.2H7 | 329929 (AB_11218984) |
| CD57 | FITC | Biolegend | HNK-1 | 359604 (AB_2562387) |
| CD14 | SparkBlue550 | Biolegend | 63D3 | 367148 (AB_2832724) |
| CD45 | PerCP | Biolegend | 2D1 | 368506 (AB_2566358) |
| CD11b | PCPCy5.5 | Biolegend | ICRF44 | 301328 (AB_10933428) |
| CD274 (PD-L1) | PE | Biolegend | 10F.9G2 | 124308 (AB_2073556) |
| CD24 | PEDz594 | Biolegend | ML5 | 311134 (AB_2566349) |
| CD95 (Fas) | PECy5 | Biolegend | DX2 | 305610 (AB_314548) |
| CD183 (CXCR3) | PECy7 | Biolegend | G025H7 | 353720 (AB_11219383) |
| CD27 | APC | Biolegend | M-T271 | 356410 (AB_2561957) |
| CD1c | AF647 | Biolegend | L161 | 331510 (AB_1186032) |
| CD19 | SparkNIR685 | Biolegend | HIB19 | 302270 (AB_2832581) |
| HLA-DR | APCF750 | Biolegend | L243 | 307658 (AB_2572101) |
| CD38 | APC-Fire810 | Biolegend | L243 | 303550 (AB_2860784) |
| CD4 | CF568 | Cytek | SK3 | SKU R7-20042 (AB_2885083) |
| CD123 | Super Bright 436 | ThermoFisher | 6H6 | 62-1239-42 (AB_2662727) |
| CD161 | eFluor450 | ThermoFisher | HP-3G10 | 48-1619-42 (AB_10854273) |
| CD20 | Pacific Orange | ThermoFisher | HI47 | MHCD2030 (AB_10375578) |
| TCRgd | PCPCeF710 | ThermoFisher | B1.1 | 46-9959-42 (AB_2573926) |
| CD25 | PE-AF700 | ThermoFisher | CD25-3G10 | MHCD2524 (AB_2539740) |

AF, AlexaFluor; BV, Brilliant Violet; BUV, Brilliant Ultra Violet; SB, super bright.

**Supplementary Table 2. List of all fluorochrome-conjugated monoclonal antibodies for flow cytometry analysis of immune-checkpoints expression on immune cell populations.**

| Marker | Fluorochrome | Source | Clone | Catalog number (RRID) |
| --- | --- | --- | --- | --- |
| CD162 (PSGL-1) | BV395 | BD | KPL-1 | 743484 (AB_2741540) |
| HLA-DR | BV496 | BD | G46-6 | 749866 (AB_2874106) |
| CD276 (B7-H3) | BUV563 | BD | 7-517 | 748380 (AB_2872799) |
| CD134 | BUV661 | BD | ACT35 | 750645 (AB_2874773) |
| VISTA (B7-H5) | BUV737 | BD | MIH65.rMAb | 749648 (AB_2873924) |
| CD108 (SEMA7A) | BUV805 | BD | KS-2 | 749451 (AB_2873819) |
| TIGIT | BV421 | Biolegend | A15153G | 372710 (AB_2632925) |
| CD1c | SB436 | ThermoFisher | L161 | 62-0015-42 (AB_2762426) |
| CD270 (HVEM) | BV480 | BD | CW10 | 746607 (AB_2743888) |
| CD16 | BV510 | Biolegend | 3G8 | 302048 (AB_2562085) |
| CD8 | Pacific Orange | ThermoFisher | 3B5 | MHCD0830 (AB_10372066) |
| CD4 | BV570 | Biolegend | RPA-T4 | 300534 (AB_2563791) |
| CD33 (Siglec-3) | BV605 | Biolegend | P67.6 | 366612 (AB_2566405) |
| CD321 (JAM-1) | BV650 | BD | M.Ab.F11 | 744066 (AB_2741969) |
| CD206 (MMR) | BV711 | Biolegend | 15-2 | 321136 (AB_2687200) |
| CD56 | BV750 | Biolegend | 5.1H11 | 362556 (AB_2801001) |
| CD137 | BV786 | BD | 4B4-1 | 741000 (AB_2740623) |
| CD14 | AF488 | Biolegend | 63D3 | 367130 (AB_2721360) |
| CD3 | SparkBlue550 | Biolegend | SK7 | 344852 (AB_2819985) |
| CD11b | PerCp | Biolegend | M1/70 | 101230 (AB_2129374) |
| CD155 | PerCp-eFluor710 | ThermoFisher | 2H7CD155 | 46-1550-42 (AB_2573720) |
| GITR-L (TNFSF18) | PE | R&D Systems | 109101 | FAB6941P (AB_2303692) |
| CD226 (DNAM-1) | PE/Dazzle™ 594 | Biolegend | 11A8 | 338318 (AB_2721694) |
| CD357 (GITR) | PE/Cyanine5 | Biolegend | 621 | 311608 (AB_2240646) |
| CD158b (KIR2DL2) | PE/Cyanine7 | Biolegend | DX27 | 312610 (AB_2563375) |
| CD170 (Siglec-5) | APC | Biolegend | 1A5 | 352006 (AB_2564262) |
| CD152 (CTLA-4) | AF647 | Biolegend | BNI3 | 369626 (AB_2832728) |
| LAG-3 (CD223) | APC-R700 | BD | T47-530 | 565774 (AB_2744329) |
| CD366 (Tim-3) | APC-eFluor780 | ThermoFisher | F38-2E2 | 47-3109-42 (AB_2637361) |

AF, AlexaFluor; BV, Brilliant Violet; BUV, Brilliant Ultra Violet; SB, super bright.

**Supplementary Table 3. List of all fluorochrome-conjugated monoclonal antibodies for flow cytometry analysis of CD4/CD8 proliferation.**

| Marker | Fluorochrome | Source | Clone | Catalog number (RRID) |
| --- | --- | --- | --- | --- |
| CD45RA | BUV395 | BD | 5H9 | 740315 (AB_2740052) |
| CD62L | BV615 | BD | SK11 | 751364 (AB_2875371) |
| CD8 | BUV805 | BD | SK1 | 612889 (AB_2833078) |
| CD197 (CCR7) | BV421 | Biolegend | G043H7 | 353208 (AB_11203894) |
| CD3 | BV510 | Biolegend | OKT3 | 317332 (AB_2561943) |
| CD28 | BV650 | Biolegend | CD28.2 | 302946 (AB_2616855) |
| CD45 | PerCP | Biolegend | 2D1 | 368506 (AB_2566358) |
| CD4 | CF568 | Cytek | SK3 | SKU R7-20042 (AB_2885083) |

BV, Brilliant Violet; BUV, Brilliant Ultra Violet.

**Supplementary Table 4. List of all fluorochrome-conjugated monoclonal antibodies for flow cytometry analysis of monocyte phenotyping.**

| Marker | Fluorochrome | Source | Clone | Catalog number (RRID) |
| --- | --- | --- | --- | --- |
| CD162 (PSGL-1) | BV395 | BD | KPL-1 | 743484 (AB_2741540) |
| HLA-DR | BV496 | BD | G46-6 | 749866 (AB_2874106) |
| CD274 (PD-L1) | PE | Biolegend | 10F.9G2 | 124308 (AB_2073556) |
| CD16 | BV510 | Biolegend | 3G8 | 302048 (AB_2562085) |
| CD33 (Siglec-3) | BV605 | Biolegend | P67.6 | 366612 (AB_2566405) |
| CD206 (MMR) | BV711 | Biolegend | 15-2 | 321136 (AB_2687200) |
| CD14 | AF488 | Biolegend | 63D3 | 367130 (AB_2721360) |
| CD11b | PerCp | Biolegend | M1/70 | 101230 (AB_2129374) |
| CD62L | BV615 | BD | SK11 | 751364 (AB_2875371) |
| CD86 | BUV737 | BD | FUN-1 | 612784 (AB_2814790) |
| CD95 (Fas) | PECy5 | Biolegend | DX2 | 305610 (AB_314548) |
| CD226 (DNAM-1) | PE/Dazzle™ 594 | Biolegend | 11A8 | 338318 (AB_2721694) |
| CD170 (Siglec-5) | APC | Biolegend | 1A5 | 352006 (AB_2564262) |

AF, AlexaFluor; BV, Brilliant Violet; BUV, Brilliant Ultra Violet; SB, super bright.

**Supplementary Table 5. Presenting symptoms and clinical findings of PAHUA and AIH patients of discovery cohort.**

| Symptom or Finding | All patients (N=21) |
| --- | --- |
|  | *no. (%)* |
| Presenting features |  |
| Fever | 7 (33) |
| Jaundice | 14 (67) |
| Vomiting | 12 (57) |
| Diarrhea | 6 (29) |
| Pale stools | 15 (71) |
| Abdominal pain | 8 (38) |
| Lethargy | 8 (38) |
| Dark urine | 15 (71) |
| Coryza | 3 (14) |
| Pruritus | 13 (62) |
| Findings on exploration |  |
| Mild hepatomegaly | 14 (67) |
| Mild splenomegaly | 6 (29) |
| Abdominal lymph nodes^*^ | 7 (33) |

^*^By ultrasound.

**Supplementary Table 6. Pathogen test in PAHUA and AIH patients and HVs.**

| Test | PAHUA (n=17)  *no. of patients with positive test (%)* | AIH (n=4)  *no. of patients with positive test (%)* | HVs (n=8)  *no. of patients with positive test (%)* |
| --- | --- | --- | --- |
| Human adenovirus PCR | 3 (18) | 0 (0) | - |
| with co-infections (Influenza A) | 2 (67) | 0 (0) | - |
| SARS-CoV-2 |  |  |  |
| Serologic test by ELISA (Spike) | 12 (71) | 3 (75) | - |
| Serologic test by LegendPlex (Spike) | 15 (88) | 4 (100) | 8 (100) |
| Serologic test by LegendPlex  (Nucleocapsid) | 15 (88) | 4 (100) | 7 (88) |
| Respiratory syncytial virus PCR | 2 (12) | 0 (0) | - |
| Norovirus or sapovirus PCR | 4 (24) | 0 (0) | - |
| Human cytomegalovirus PCR | 1 (6) | 0 (0) | - |
| Herpes virus PCR | 2 (12) | 0 (0) | - |
| Influenza type A or B PCR | 10 (29) | 0 (0) | - |

**Supplementary Table 7. Differentially expressed variables in PAHUA vs. HVs, PAHUA vs. AIH and AIH vs. HV comparisons.**

| Upregulated variables | | |
| --- | --- | --- |
|  | | |
| PAHUA *vs*. HV | **PAHUA *vs*. AIH** | **AIH *vs*. HV** |
|  | | |
| Gal-9* | % Intermediate Effector EM CD8 cells | % Naïve CD8^+^ cells on non^-^proliferative unstimulated cells |
| % CD256 on NK cells | % CD27^+^CD95^-^ NKT CD4^-^CD57^-^ cells | % Naïve CD8^+^ cells on non^-^proliferative PWD stimulated cells |
| % EM CXCR3^+^CD95^+^ Tregs | sCD27 | % Early like Effector EM CD8^+^ cells |
| sCD25 | % CM CXCR3^+^CD95^+^ Tregs | MFI CD11b on CD14^+^ cells unstimulated |
| % EM CD27^-^CD28^-^ CD4^-^CD8^-^ T cells | % EMRA CXCR5^-^CCR6^-^ CD4^+^CD8^+^ T cells | % CD27^-^CD95^+^ NKT CD4^-^CD57^-^ cells |
| % CD33 on CD8 cells | % CXCR5^-^CD95^+^ Switched B cells | % SIGLEC5 on NK cells |
| % EMRA CD27^-^CD28^-^ CD4^+^CD8^+^ T cells^#^ | % Early like effector EM CD57^-^CD95^-^ CD8 cells | % OX-40 on CD4^+^ cells |
| % Early Effector EM CD57^-^CD95^-^ CD8 cells* | % CD206 on NKT cells | MFI CD11b on CD14^+^ cells LPS stimulated |
| % CM CD57^-^CD95^-^ CD8 cells | % CD38^-^CD24^-^ Naïve B cells | % CM CD57^+^CD95^+^ CD4^-^CD8^-^ T cells |
| % Tim-3 on NK cells | % EMRA CD57^-^CD95^-^ CD4^+^CD8^+^ T cells | % EM CD57^+^CD95^+^ CD4^-^CD8^-^ T cells |
| % B7H5 on CD8 cells | Gal-9* | sCTLA-4 |
| % EM CXCR3^+^CD95^-^ Tregs | % CXCR5^-^CD95^-^ CD27^-^IgD^-^ B cells | % EMRA CD27^-^CD28^-^ CD4^+^CD8^+^ T cells^#^ |
| % CD57^-^CD95^+^ NK2 cells | % Early Effector EM CD57^-^CD95^-^ CD8 cells* | % CD57^+^CD95^+^ NK6 cells |
| sTim-3* | % Naïve CXCR5^-^CCR6^-^ CD4^+^CD8^+^ T cells | % CD57^+^CD95^+^ NK3 cells |
| % CD270 on CD4 cells | % CCR6^+^CD161^-^ NKT CD8^-^CD57^+^ cells | % CD57^-^CD95^+^ NKdim cells |
|  | % CXCR5^-^CD95^-^ Unswitched B cells | % CM CXCR5^+^CCR6^+^ CD4^+^CD8^+^ T cells |
|  | % CXCR5^-^CD95^-^ Naïve B cells | % CM CXCR5^+^CCR6^-^ CD4^+^CD8^+^ T cells |
|  | sTim-3* |  |
|  | % EM CD57^+^CD95^-^ CD4^-^CD8^-^ T cells |  |
|  | % Terminal Effector EMRA CD57^+^CD95^-^ CD8^+^ T cells |  |
|  | % EM CXCR5^-^CCR6^-^ CD4^+^CD8^+^ T cells |  |
|  | % CD57^-^CD95^-^ NKdim cells |  |
|  | % Intermediate effector EM CD57^+^CD95^-^ CD8 cells |  |
|  | % EM CD57^-^CD95^-^ CD4^-^CD8^-^ cells |  |
|  | % CM CD57^-^CD95^-^ CD4^-^CD8^-^ cells |  |
|  | % PD-L1^+^ on CD14^+^ cells B^-^glucan stimulated |  |
|  | % cDC1 on CD45^+^ cells |  |
|  | % PD-L1^+^ on CD14^+^ cells R848 stimulated |  |

*, shared variables between PAHUA *vs.* HV and PAHUA *vs.* AIH comparisons; #, shared variables between PAHUA *vs.* HV and AIH *vs.* HV comparisons

| Downregulated variables | | |
| --- | --- | --- |
|  | | |
| PAHUA *vs*. HV | **PAHUA *vs*. AIH** | **AIH *vs*. HV** |
|  | | |
| % CD158b on CD8 cells | % EMRA CD57^+^CD95^+^ CD4^+^CD8^+^ T cells | % Naïve CXCR5^-^CCR6^+^ CD4^+^CD8^+^ T cells |
| % Naïve CD27^-^CD28^-^ CD4^-^CD8^-^ T cells | Spike Protein RBD | % PD-L1^+^ on CD14^+^ cells LPS stimulated |
| % CD108 on CD8 cells | % CM CXCR5^+^CCR6^-^ CD4^+^CD8^+^ T cells | % CM CD57^-^CD95^-^ CD4^-^CD8^-^ T cells |
| % CD223on CD8 cells | % Naïve CD161^+^CD38^-^ CD4^+^CD8^+^ T cells | % CD57^-^CD95^-^ NKdim cells |
| % CD357 on CD8 cells | % CM CXCR5^+^CCR6^+^ CD4^+^CD8^+^ T cells | % EMRA CD8^+^ cells on non^-^proliferative unstimulated cells |
| % CD8^+^ on CD3 cells | % CD56^+^CD57^+^ TCRgd cells | % EM CD57^-^CD95^-^ CD4^-^CD8^-^ T cells |
| % Terminal Effector EMRA CD57^-^CD95^+^ CD8 cells | % Intermediate effector EM CD57^+^CD95^+^ CD8 cells | % CM CXCR5^-^CCR6^-^ CD4^+^CD8^+^ T cells |
| % EM CD161^+^CD38^-^ CD4^-^CD8^-^ T cells | IL-1β | % EM CXCR5^-^CCR6^-^ CD4^+^CD8^+^ T cells |
|  | MFI CD11b on CD14^+^ cells LPS stimulated | % EMRA CD8^+^ cells on non^-^proliferative PWD stimulated cells |
|  | % IgG^+^ B cells | % EM CD27^+^CD28^-^ CD4^+^CD8^+^ T cells |
|  | IL-2 | % Naïve CXCR5^-^CCR6^-^ CD4^+^CD8^+^ T cells |
|  | MFI CD11b on CD14^+^ cells unstimulated | % CD38^-^CD24^-^ Naïve B cells |
|  | % OX40 on NK cells | % EMRA CD57^-^CD95^-^ CD4^+^CD8^+^ T cells |
|  | % Early like Effector EM CD4 cells | % EMRA CD27^+^CD28^-^ CD4^+^CD8^+^ T cells |
|  | % EM CD4 cells | % CD57^-^CD95^-^ NK3 cells |
|  | MIF CD11b on CD14^+^ cells B^-^glucan stimulated | % CD321 on NKT cells |
|  | % CM Tregs | % EMRA CXCR5^-^CCR6^-^ CD4^+^CD8^+^ T cells |
|  | % EM CD27^+^CD28^+^ CD4^-^CD8^-^ T cells | % Intermediate Effector EM CD8 cells |
|  | % CD206 on NK cells |  |
|  | % CM CD57^-^CD95^+^ CD4^-^CD8^-^ T cells |  |
|  | MFI CD33 on CD14^+^ cells unstimulated |  |
|  | MFI SIGLEC5 on CD14^+^ cells unstimulated |  |
|  | MFI CD33 on CD14^+^ cells R848 stimulated |  |
